# Reprogramming Cdr2-Dependent Geometry-Based Cell Size Control in Fission Yeast

**DOI:** 10.1016/j.cub.2018.12.017

**Published:** 2019-01-21

**Authors:** Giuseppe Facchetti, Benjamin Knapp, Ignacio Flor-Parra, Fred Chang, Martin Howard

**Affiliations:** 1 Computational and Systems Biology, John Innes Centre, Norwich, UK; 2 Department of Cell and Tissue Biology, University of California, San Francisco, San Francisco, CA, USA; 3 Centro Andaluz de Biología del Desarrollo, Universidad Pablo de Olavide/CSIC/Junta de Andalucia, Seville, Spain

**Keywords:** cell size homeostasis, sizer control, fission yeast, Cdr2, synthetic biology

## Abstract

How cell size is determined and maintained remains unclear, even in simple model organisms. In proliferating cells, cell size is regulated by coordinating growth and division through sizer, adder, or timer mechanisms or through some combination [1, 2]. Currently, the best-characterized example of sizer behavior is in fission yeast, *Schizosaccharomyces pombe*, which enters mitosis at a minimal cell size threshold. The peripheral membrane kinase Cdr2 localizes in clusters (nodes) on the medial plasma membrane and promotes mitotic entry [3]. Here, we show that the Cdr2 nodal density, which scales with cell size, is used by the cell to sense and control its size. By analyzing cells of different widths, we first show that *cdr2*^+^ cells divide at a fixed cell surface area. However, division in the *cdr2*Δ mutant is more closely specified by cell volume, suggesting that Cdr2 is essential for area sensing and supporting the existence of a Cdr2-independent secondary sizer mechanism more closely based on volume. To investigate how Cdr2 nodes may sense area, we derive a minimal mathematical model that incorporates the cytoplasmic kinase Ssp1 as a Cdr2 activator. The model predicts that a *cdr2* mutant in an Ssp1 phosphorylation site (*cdr2-T166A*) [4] should form nodes whose density registers cell length. We confirm this prediction experimentally and find that thin cells now follow this new scaling by dividing at constant length instead of area. This work supports the role of Cdr2 as a sizer factor and highlights the importance of studying geometrical aspects of size control.

## Highlights

• Cdr2 nodal density mediates surface-area-based size control in fission yeast
• Mathematical modeling predicts length scaling for nodal mutant Cdr2-T166A density
• Thin *cdr2-T166A* cells divide using length, demonstrating size control reprogramming
• A secondary sizer mechanism based more closely on volume is active in a *cdr2* mutant

## Graphical Abstract

**Figure:**
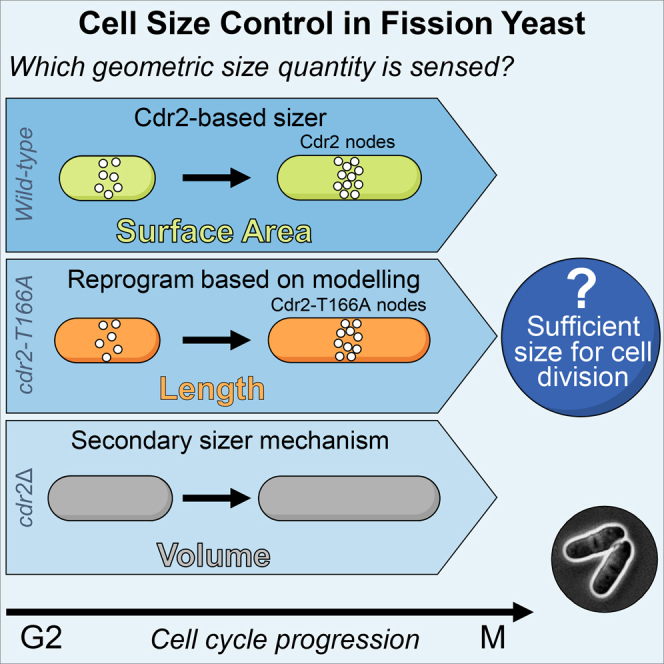

## Teaser

By using cells of different widths, Facchetti et al. show that fission yeast size homeostasis is based on cell surface area as registered by Cdr2 nodal density. A mathematical model allows reprogramming of this Cdr2-based size regulation from area to length. Secondary size control in a *cdr2* mutant more closely based on volume is also uncovered.

## Results and Discussion

A variety of strategies are believed to be used for cell size control and homeostasis. In sizer behavior, cells grow to a minimal cell size threshold before committing to division. In adder behavior, cells grow a fixed size increment, regardless of initial size, a strategy that has been observed in various bacteria, budding yeast, and mammalian cells [5, 6, 7]. The rod-shaped fission yeast *S. pombe* exhibits sizer behavior [8], where cells grow during interphase to a target size of 14 ± 1 μm in length before entering mitosis and dividing medially. Recently, evidence has emerged for multiple layers of size control operating within the cell cycle. For example, in budding yeast, different regimes in different cell phases, including sizer control at the G1/M transition, may account for adder-like behavior over the whole cell cycle [9, 10]. Furthermore, some fission yeast mutants exhibit two-layer size control with sizer and adder timer behaviors [11]. However, even for simple sizer behavior, a key question remains how and what aspect of cell size is sensed and how this information is transduced to the cell cycle control machinery.

In fission yeast, a leading candidate sizer protein is Cdr2, a SAD protein kinase [3, 4, 12]. Cdr2 may be part of an activator accumulation mechanism, which triggers mitosis when Cdr2 activity exceeds a threshold [3]. Cdr2 regulates cell size and mitotic entry by activating Cdk1 through Wee1 inhibition [13, 14]. Cdr2 is a peripheral membrane protein that binds to the plasma membrane and accumulates in discrete clusters on the plasma membrane (“nodes”), which form a broad band around the nucleus. These nodes contain at least 7 other proteins, including those involved in cytokinesis and cell cycle control, including Wee1 and Cdr1 [15, 16]. Although the nodes are generally stable structures, individual Cdr2 molecules and other node proteins dynamically exchange between the nodes, membrane, and cytoplasm [3, 17]. These nodes have been proposed as an important element in cell size control, as their number scales with cell size, and *cdr2* mutants defective in node association are defective in size control [3, 18]. Recent studies have suggested that the Cdr1 and Cdr2 kinases in the nodes transiently recruit and inactivate Wee1 by phosphorylation [19, 20]. Upstream Cdr2 regulators include an inhibitory kinase Pom1 [21, 22] and an activating kinase CaMK Ssp1 [4, 23]. Pom1 binds to the plasma membrane and is enriched at cell tips [24, 25], whereas Ssp1 is cytoplasmic and activates Cdr2 kinase activity by T166 phosphorylation in the Cdr2 kinase domain [4]. Here, we show that Cdr2 nodes play a critical role in sensing cell surface area for size control and that, as predicted by mathematical modeling, a mutation in Cdr2 can reprogram the cells to instead sense cell length.

### Fission Yeast Size Homeostasis Is Based on Surface Area Sensing

**Figure 1:**
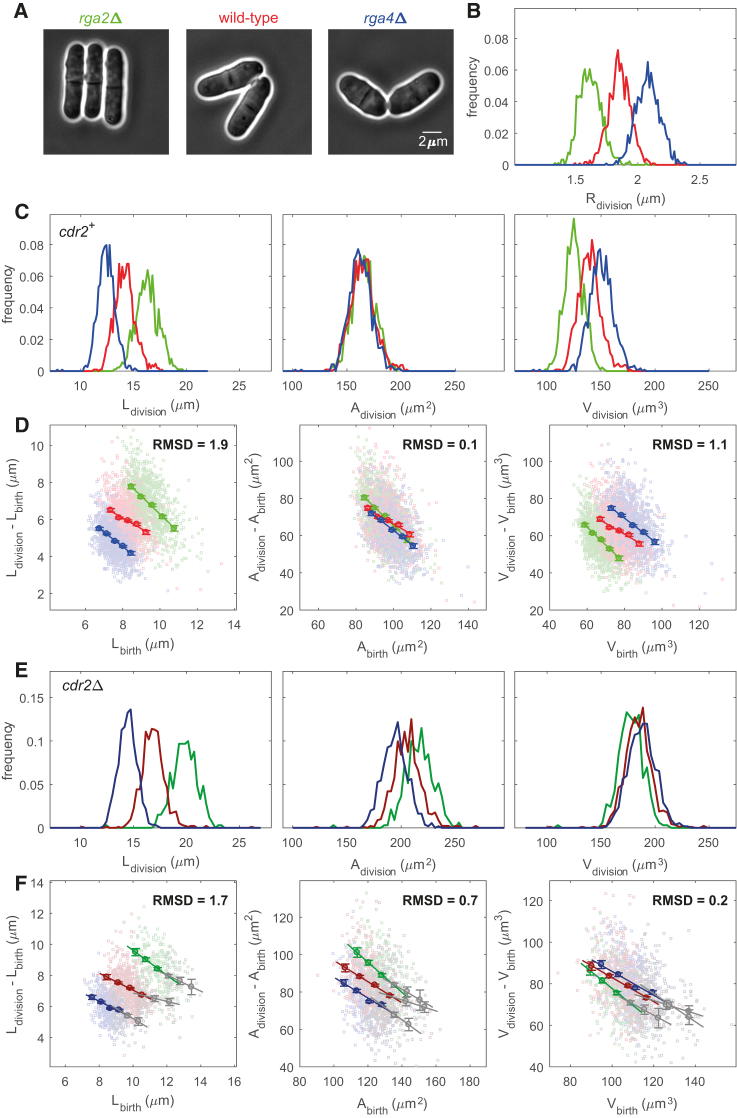
Cdr2 Is Required for Surface-Area-Based Cell Size Control (A) Wild-type cells enter mitosis when they reach a specific surface area. Phase contrast images of representative *rga2*Δ (thin mutant), wild-type (normal width), and *rga4*Δ (fat mutant) cells at division are shown. These cells of different widths divide at different lengths. Scale bar: 2 μm. (B) Distribution of cell radius (*R*) at division for *rga2*Δ, wild-type, and *rga4*Δ. (C) Distribution of cell length (*L*), surface area (*A*), and volume (*V*) at division for *rga2*Δ, wild-type, and *rga4*Δ. (D) Size homeostasis plots using cell length, surface area, or volume as size measure for *rga2*Δ, wild-type, and *rga4*Δ. Slopes are −0.9, −0.6, and −0.7, respectively. (E and F) *cdr2Δ* cells enter mitosis approximately at a specific volume. Distributions at division (E) and size homeostasis plots (F) for *cdr2*Δ *rga2*Δ, *cdr2*Δ, and *cdr2*Δ *rga4*Δ. Slopes for the data from shorter cells (less than about 60% of the average division length, see Figures S1C and S1D) are −0.8, −0.6, and −0.6, respectively (colored lines in F). Slopes for the data from longer cells are −0.4, −0.3, and −0.6, respectively (gray lines in F). Color legend: *rga2*Δ (FC2947; green; n = 892), wild-type (FC15; red; n = 1,061), and *rga4*Δ (FC1901; blue; n = 1,173); *cdr2*Δ *rga2*Δ (FC3225; dark green; n = 507), *cdr2*Δ (FC3161; dark red; n = 1,277), and *cdr2*Δ *rga4*Δ (FC3227; dark blue; n = 984). Binned data (with mean value ± SE) and associated regression line are shown in (D) and (F). Normalized root-mean-square deviation (RMSD between binned data; STAR Methods) is also stated. t tests on normalized RMSDs all give p values < 10^−100^ (D) or < 10^−20^ (F). See also Figure S1.

For sizer mechanisms, an outstanding question is whether cells sense their size by monitoring volume, surface area, length, or some other geometric quantity. As wild-type fission yeast cells are rods of approximately constant width, both surface area ($A_{memb}=2\pi RL$, with R and L the cell radius and length, respectively) and volume $\left(V_{cell}\approx \pi R^{2}L\right)$ approximately scale with length. To distinguish between length, area, or volume homeostasis, we analyzed mutants with altered cell radius. We used the RhoGAP mutants *rga2*Δ and *rga4*Δ, which form thin and fat rods, respectively, but have otherwise intact cell shape [26, 27, 28] (Figures 1A and 1B). Growth rate is also preserved in these mutants, with less than 5% variability between strains. Previous data using these mutants suggested that fission yeast cells divide at a constant surface area [3]. Here, we exploited recent technical advances using cells grown in microfluidic chambers with constant media flow and temperature control, as well as automated machine-learning image analyses methods, including sub-pixel resolution segmentation [29, 30], to acquire datasets with much larger sample sizes and reduced biases (STAR Methods). We confirmed with a large dataset (n = 3,126) that cells with a range of widths divided at the same surface area (≈165 μm^2^), but not the same length or volume (Figure 1C).

We next tested whether fission yeast senses surface area for size homeostasis. We imaged growing cells by time-lapse microscopy; measured the length, surface area, and volume of cells at the beginning and end of the cell cycle; and then plotted how much these quantities increased from birth to division against birth size [1, 2]. For a sizer mechanism, such a plot should show a slope of −1 [2, 8]. Previous analyses used length as the size measure [8, 11, 31]. In the same way, we first verified that the sizer mechanism is preserved in the *rga2*Δ and *rga4*Δ mutants: plots show a slope close to −1 for each single strain (Figure 1D). We expect the strains to show the same behavior only if the plot is based on the geometric feature that is actually used for size control. As the size data did not overlap when length was used as the size measure, we made the same plot using surface area and volume. Among the three geometry quantities, surface area provided the tightest data overlap from the three strains (as measured by a lower normalized root-mean-square deviation [RMSD] of binned data; STAR Methods; Figure 1D). Statistical tests on the difference between the RMSDs showed p values < 10^−100^ (STAR Methods). All the above conclusions were robust to the methodology used to calculate area and volume (Figure S1A). Moreover, by analyzing the data with $R^{\gamma }L$ as a generalized and unbiased cell size measure (where γ can vary continuously; STAR Methods), the smallest RMSD is achieved for γ≈1 (Figure S1B), again confirming surface area sizing.

### Deletion of *cdr2* Disrupts Surface-Area-Based Size Homeostasis

Previous work had implicated Cdr2 as a candidate sizer molecule [3]. *cdr2*Δ mutant cells are viable and exhibit a similar cell width as the wild-type but divide at longer lengths [12, 32]. Although cell size has been shown to be sensitive to Cdr2 dosage [3, 12, 33], the *cdr2*Δ mutant has not previously been tested for size homeostasis. As has been seen with some other mutants, such as *pom1*Δ and a strain expressing the Cdc13-L-Cdc2 fusion protein [11], the size homeostasis plots for *cdr2*Δ mutants were best fit with lines with two slopes, indicative of different regimes at different sizes. The first had a −0.6 slope for cells with a birth size below 10.5 μm (∼60% of the average division length; Figures S1C and S1D), consistent with a sizer mechanism for cells born at a smaller size. The second had a −0.3 slope, closer to adder- or timer-like behavior for cells born at a larger size. Thus, *cdr2*Δ cells with shorter birth sizes are still capable of sizer behavior.

We next investigated whether this *cdr2*Δ sizer mechanism is still area based. We varied the cell radius of the *cdr2*Δ cells by constructing double mutants with *rga2* and *rga4* deletions and analyzed cells in the sizer regime (i.e., smaller birth sizes). Compared to *cdr2*^+^ strains, these cells divided more closely aligned to volume (at ≈180 μm^3^; Figure 1E), rather than at a specific area. Similarly, size homeostasis plots showed the smallest RMSD when volume was used as the geometrical quantity (Figures 1F, S1E, and S1F for a repeated experiment; p values < 10^−20^). Results were robust to changes in the area or volume calculation methodology (Figure S1G) and to analysis with the unbiased size measure $R^{\gamma }L$ (smallest RMSD for γ≈1.6; close to the theoretical value for volume of ≈1.75; Figure S1H; STAR Methods). Therefore, *cdr2* deletion causes a loss of area-based size control, leading instead to cell size regulation potentially through a secondary sizer mechanism more closely based on volume. This mechanism has a division size coefficient of variation as low as the wild-type (≈7.5%), suggesting precise sizer control. Such a secondary sizer could also explain a size homeostasis slope ≈ −1 in mutants lacking Cdk1-Tyr15 phosphorylation [11, 34], the output of the Cdr2/Wee1 pathway.

### Cdr2 Nodal Density Scales with Surface Area

**Figure 2:**
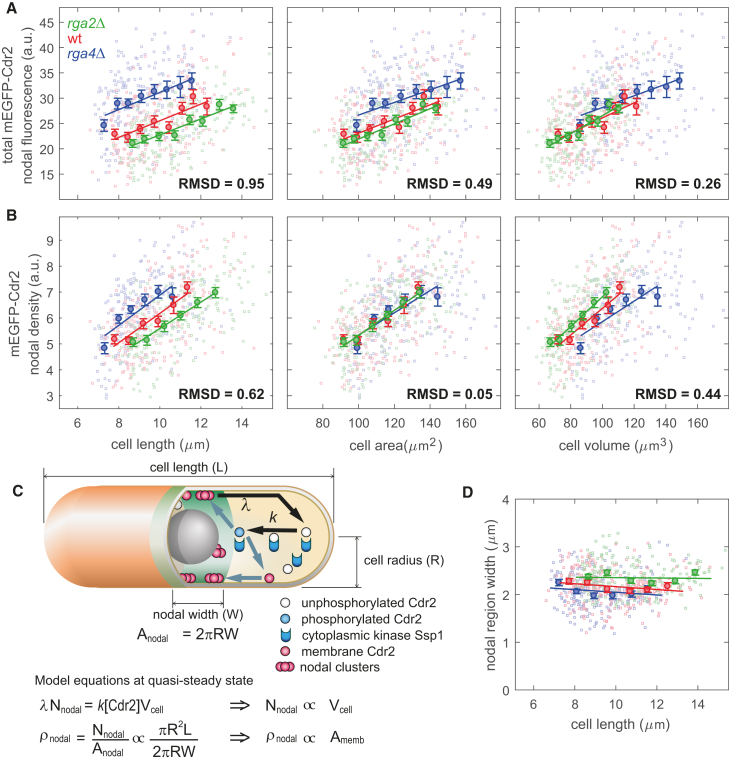
Density of Cdr2 in the Nodal Region Scales with Cell Surface Area, in Agreement with Results from Mathematical Model (A) Plots of total nodal intensity of mEGFP-Cdr2 for *rga2*Δ, wild-type, and *rga4*Δ strains as function of length, surface area, and volume. (B) Plots of nodal density of mEGFP-Cdr2, otherwise as in (A). (C) Sketch and equations of minimal model to explain Cdr2 nodal density scaling with cell surface area in wild-type (STAR Methods). Symbols: [Cdr2], cytoplasmic concentration of Cdr2; $N_{nodal}$, total nodal amount (fluorescence) of Cdr2; $\rho_{nodal}$, nodal density of Cdr2;k, kinetic parameter of Cdr2 phosphorylation by cytoplasmic Ssp1; λ, kinetic parameter of Cdr2 dissociation from nodes; $V_{cell}$, cell volume;$\;A_{memb}$, total membrane surface area; $A_{nodal}$, nodal area with constant width W (see D). (D) Plot of nodal region width as function of cell length for *rga2*Δ, wild-type, and *rga4*Δ. Color legend: *rga2*Δ (FC3187; green; n = 211), wild-type (FC3156; red; n = 224), and *rga*4Δ (FC3189; blue; n = 201). Binned data (with mean value ± SE) and associated regression line are also shown in (A), (B), and (D). Normalized RMSD (between binned data; STAR Methods) also stated in (A) and (B): t tests on normalized RMSDs all give p values < 10^−15^. See also Figure S2.

Together, these findings imply that Cdr2 plays a critical role in area sensing. We hypothesized that some relevant property of Cdr2 used in size control would scale with area. We therefore investigated which Cdr2 property showed area scaling and tested whether this scaling relationship is critical for size control. We first measured Cdr2 protein concentrations, using fluorescence intensity measurements in cells expressing mEGFP-Cdr2 from the endogenous chromosomal locus [3]. The total Cdr2 and cytoplasmic Cdr2 concentrations did not vary significantly with cell length (Figures S2A and S2B) [3, 12]. We also confirmed that the total Cdr2 nodal intensity (total amount of Cdr2 in the nodal region; STAR Methods) increases with cell size, as shown previously [3, 18, 33, 35]. Similarly, the Cdr2 nodal density (amount of Cdr2 per unit area in the nodal region; STAR Methods) increases with cell size. By plotting the total Cdr2 nodal fluorescence and nodal density as a function of cell length, area, and volume in the wild-type, *rga2*Δ, and *rga4*Δ strains, we found using normalized RMSDs that total Cdr2 nodal intensity scaled with cell volume, whereas Cdr2 nodal density scaled with total cellular surface area (Figures 2A and 2B; p values < 10^−15^; discussion of the x axis overlaps in STAR Methods). Analysis using an unbiased expression for cell size confirmed these scalings (Figures S2C and S2D). The mean width of the Cdr2 nodal area was roughly independent of cell radius and remained constant in cells of different lengths (Figure 2C). Our results were robust to changes in the area or volume calculation methodology (Figures S2E and S2F), and a repeat independent experiment yielded similar results (Figures S2G and S2H). These measurements suggest that the Cdr2 density in the nodal region could be a critical quantity used to monitor cell size.

### Mathematical Modeling Predicts that the Cdr2 Nodal Density Scales with Cell Length in the *cdr2-T166A* Mutant

**Figure 3:**
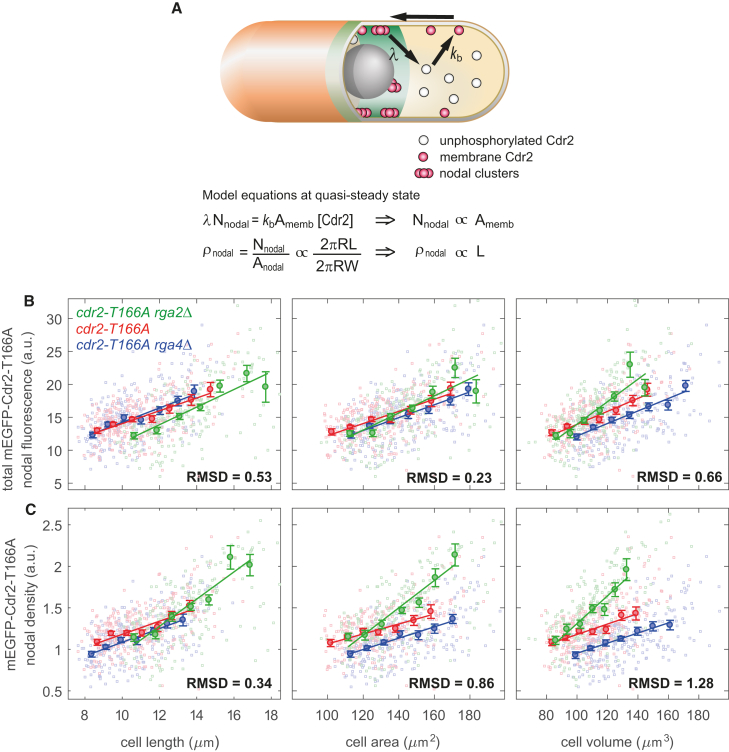
Model Prediction and Validation of Altered Geometrical Scaling for Nodal Cdr2-T166A Levels (A) Schematic of the model for *cdr2-T166A* mutant (STAR Methods). Equations predict length scaling of Cdr2-T166A nodal density. Symbols are as in Figure 2C, with the addition of $k_{b}$ as binding constant of unphosphorylated Cdr2 to cell membrane. (B) Plots of total nodal fluorescence of Cdr2-T166A for *cdr2-T166A rga2*Δ, *cdr2-T166A*, and *cdr2-T166A rga4*Δ as function of length, surface area, and volume. (C) Plots of nodal density of Cdr2-T166A, otherwise as in (B). Color legend: *cdr2-T166A rga2*Δ (FC3180; green; n = 150), *cdr2-T166A* (FC3164; red; n = 151), and *cdr2-T166A rga4*Δ (FC3183; blue; n = 140). Binned data (with mean value ± SE) and associated regression line are shown in (B) and (C). Normalized RMSD (between binned data; STAR Methods) is also stated in (B) and (C); t tests on normalized RMSDs all give p values < 10^−5^. See also Figure S3.

We next sought to see whether we could reprogram cell size sensing by changing properties of the Cdr2 protein. To help with this investigation, we developed a simple mathematical model of the Cdr2 nodal scaling (Figures 2C and 2D; STAR Methods). Compared to previous analysis, the proposed model specifically considers Cdr2 activation by the cytoplasmic kinase Ssp1 [4, 23]. This systematic mathematical modeling approach allowed us to understand how size sensing occurs. Other models along similar lines are, of course, possible, but the model developed generated strong, verifiable predictions. Following nodal unbinding, if Cdr2 next interacts in the cytoplasmic volume through Ssp1-mediated phosphorylation, then, by balancing fluxes through the pathway, we find that the total Cdr2 nodal intensity in the model scales with cell volume. However, if, in the absence of such phosphorylation, the next interaction occurs on the membrane (binding), then flux balance forces the total Cdr2 nodal intensity in the model to scale with total cellular surface area. The Cdr2 nodal density is the total nodal intensity divided by the nodal area. A key element of the model is the restricted region occupied by the nodes at the medial cell cortex. This region has approximately fixed width (Figure 2D), causing the nodal area to scale with the cell radius. As a result, the Cdr2 nodal density should scale as the ratio between volume and radius (i.e., total cellular surface area) or as the ratio between total cellular surface area and radius (i.e., cell length), respectively (Figures 2C and 3A; STAR Methods). Assuming the nodal Cdr2 density is the basis for size control, we therefore predicted that the phosphorylation-deficient cells should divide at a specific cell length instead of a specific total membrane surface area.

To test this model prediction, we focused on a Cdr2-T166A mutant in an Ssp1-dependent phosphorylation site located at the active site of the Cdr2 kinase domain. This alteration is more specific than an *ssp1*-null allele, which has pleiotropic consequences as Ssp1 has many targets [27]. This *cdr2-T166A* allele, which has been previously characterized, is thought to be deficient in kinase activity, as the mutant cells divide at the same elongated length as a Cdr2 kinase-dead allele *cdr2-E177A* but still retains kinase-independent functions [4]. Nevertheless, there is no information available about the size control implemented by this mutant (i.e., if it is still a sizer and which geometrical sensing it might use). We therefore expressed mEGFP-Cdr2-T166A as the only Cdr2 protein in strains of different widths. As shown previously, mEGFP-Cdr2-T166A still localizes to medial nodes [4]. We also verified that the nodal region width and Cdr2-T166A cytoplasmic concentration only varied weakly with cell length and radius (Figures S3A–S3C). Strikingly, the experimental data confirmed our theoretical prediction: the total Cdr2-T166A nodal intensity scaled with total cellular surface area, with the Cdr2-T166A nodal density scaling with cell length (Figures 3B and 3C; p values < 10^−5^; unbiased cell size analysis in Figures S3D and S3E). Accordingly, images showed the Cdr2-T166A nodal intensity was visibly higher in thinner cells than fatter cells of the same volume (Figures S3F and S3G). These results were robust to changes in the area or volume calculational methodology (Figures S3H and S3I). Overall, these findings support our proposed Cdr2 nodal density size scaling mechanism.

### Length Sensing Is Implemented in Thin *cdr2-T166A* Mutant Cells

**Figure 4:**
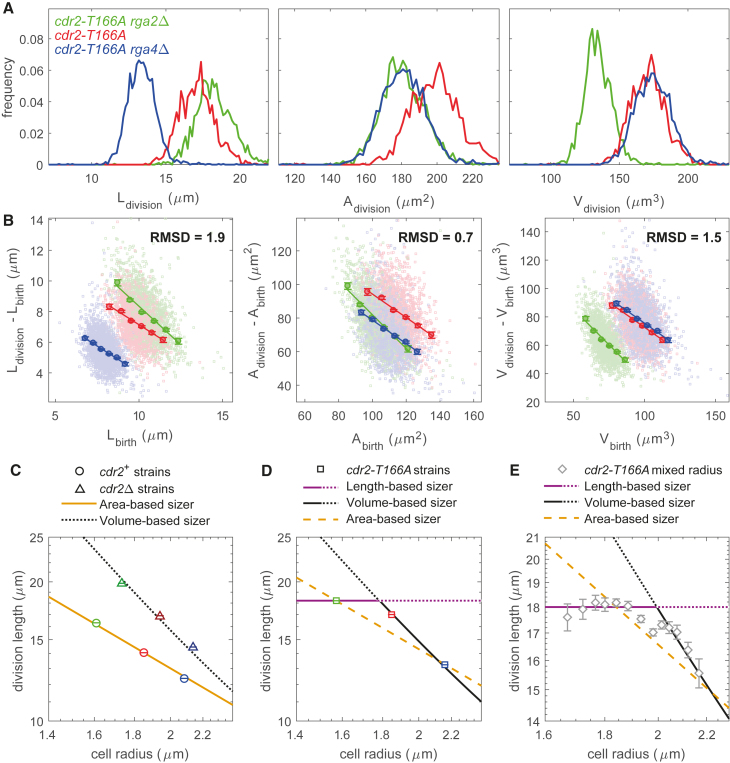
Size Homeostasis Is Based on Length in Thin *cdr2-T166A* Cells (A) Distribution of cell length, surface area, and volume at division for *cdr2-T166A rga2*Δ, *cdr2-T166A*, and *cdr2-T166A rga4*Δ. (B) Size homeostasis plots for *cdr2-T166A rga2*Δ, *cdr2-T166A*, and *cdr2-T166A rga4*Δ using cell length, surface area, or volume as size measure. Slopes are −0.9 (*cdr2-T166A rga2*Δ), −0.7 (*cdr2-T166A*), and −0.7 (*cdr2-T166A rga4*Δ). Color legend: *cdr2-T166A rga2*Δ (FC3218; green; n = 1,785), *cdr2-T166A* (FC3216; red; n = 1,561), and *cdr2-T166A rga4*Δ (FC3220; blue; n = 2,309). Binned data (with mean value ± SE) and associated regression line are shown in (B). Normalized RMSD (between binned data; STAR Methods) is also stated. (C) Relationship between division length and cell radius depends on the geometrical sensing: slope −1 for an area-based sizer mechanism (yellow full line; $\log\;L_{div}=\log\left(A_{div}/2\pi \right)-\log\;R$, with $A_{div}=165\;\mu m^{2}$), slope of ≈ −2 for a volume-based sizer (black dotted line; $\log\;L_{div}\approx \log\left(V_{div}/\pi \right)-2\;\log\;R$, with $V_{div}=180\;\mu m^{3}$). Data from Figure 1 are reported as mean value ± SE (color legend: *rga2*Δ, green; wild-type, red; *rga4*Δ, blue). Fitting is obtained by changing the intercept only (specified by $A_{div}$ or $V_{div}$). (D) Data from (A), reported as mean value ± SE (*cdr2-T166A* strains; same color legend as in A), consistent with a length-based sizer for thin cells (purple full and dotted line) and volume-based sizer for fat cells (black full and dotted line). An area-based sizer (dashed yellow line) is less consistent with the data. (E) Experiment at higher pixel resolution on a mixture of *cdr2-T166A rga2*Δ, *cdr2-T166A*, and *cdr2-T166A rga4*Δ cells (total n = 401), using bin analysis based on cell radius (with mean value ± SE), demonstrating crossover from length-based sizer (purple full and dotted line) to volume-based sizer (black full and dotted line) behavior. A single area-based sizer (dashed yellow line) cannot fit the data (p value < 10^−20^ using χ^2^ test). See also Figure S4.

We next tested whether *cdr2-T166A* cells follow the Cdr2-T166A nodal density scaling with length to divide at a specific length instead of area. We performed size homeostasis experiments on strains of different widths expressing untagged Cdr2-T166A: *cdr2-T166A rga2*Δ; *cdr2-T166A*; and *cdr2-T166A rga*4Δ (Figures 4A, 4B, S4A, and S4B). *cdr2-T166A* cells were still able to regulate their size (with size homeostasis slopes below −0.7; Figure 4B), indicating that a sizer mechanism is still operating. However, geometric analysis of the division size of the three mutants was hard to interpret (Figures 4A and S4A). Although size homeostasis RMSDs showed a minimum for surface area (RMSD = 0.7; Figures 4B and S4B), this minimal value was still higher than any previous minimal value (by more than two-fold), potentially suggesting a more complex situation. Moreover, area sensing cannot explain why *cdr2-T166A* and *cdr2-T166A rga*4Δ divided at the same volume (Figures 4A and S4A), a conclusion also supported by our size homeostasis plots (Figures 4B and S4B) and confirmed by robustness (Figure S4C) and unbiased γ analysis (Figure S4D).

To better interpret these data, we plotted log(division length) against log(cell radius). If the cells sense length, strains with different radii will fall on a flat line $\left(\log\;L_{div}=constant\right)$; for area sensing, the slope will be −1 $\left(\log\;L_{div}=\log\left(A_{div}/2\pi \right)-\log\;R\right)$ and for volume sensing, the slope will be ≈ −2 $\left(\log\;L_{div}\approx \log\left(V_{div}/\pi \right)-2\;\log\;R\right)$. Figure 4C reports the data from Figure 1: this plot supports the hypothesis that, in the presence of Cdr2, size control is based on area sensing (yellow line), which intervenes at smaller sizes before Cdr2-independent control, more closely based on volume (black dotted line), could act as a secondary sizer mechanism.

We next applied the same analysis to *cdr2-T166A* (Figure 4D). Unlike for *cdr2*^+^ and *cdr2*Δ, the data are no longer well fitted by any single line, demonstrating that size control is more complex in *cdr2-T166A*. We therefore explored the hypothesis that *cdr2-T166A* displays more than one type of geometric size control. We supposed that thinner *cdr2-T166A rga2*Δ cells divide according to length (specified by the nodal Cdr2-T166A density), although wider *cdr2-T166A* and *cdr2-T166A rga*4Δ cells divide more closely based on volume, as suggested by Figures 4A, 4B, and S4A–S4D. As shown in Figures 4D and S4B (black and purple full and dotted lines), we find that this hypothesis is indeed consistent with our data. This hypothesis predicts that higher resolution radius data (allowing more radii bins) should show a flat slope for thinner cells crossing over for wider cells to a slope ≈ −2. To test this hypothesis, we acquired higher pixel resolution images from a mixture of the same three strains. The data (Figure 4E) clearly exhibited these two slopes (i.e., length- and volume-based sizers), supporting our interpretation. Note that these data do not support potential area sensing (yellow dashed line in Figures 4D and 4E; p value < 10^−20^ using χ^2^ test) or a combination of length- and area-based sizers. With our interpretation, the area overlap between *cdr2-T166A rga2*Δ and *cdr2-T166A rga4*Δ cells (Figures 4A, 4B, S4A, and S4B) is an indirect consequence of length sensing in *cdr2-T166A rga2*Δ and sensing more closely based on volume in *cdr2-T166A rga4*Δ.

The Figure 4E data may also explain why wider *cdr2-T166A* cells divide more closely with volume not length. In these cells, the secondary Cdr2-independent mechanism is presumably still present. For wider cells, the normally secondary volume threshold for the Cdr2-independent pathway (full black line) may be attained first as the cells grow before the length threshold (dotted purple line), although for thinner cells, the length threshold (purple full line) may be reached before the volume threshold (dotted black line). It is also formally possible that *cdr2-T166A rga2*Δ cells divide according to length for some other reason associated with *rga2* deletion, though our *rga2*Δ results for *cdr2*^*+*^ and *cdr2*Δ argue against this possibility.

### Conclusions

Here, we show that fission yeast cells possess a Cdr2-dependent sizer mechanism that controls division at a specific total cellular surface area. We derive a mathematical model showing how Cdr2 may sense surface area through dynamic exchange of Cdr2 molecules between nodes, cytoplasm, and plasma membrane. Aided by this model, we find that a simple point mutation *cdr2-T166A* changes the scaling of Cdr2-T166A nodal density to cell length instead of total cellular surface area. A subset of these cells now divided at a specific cell length, supporting the key role of Cdr2 as a geometric sizer molecule. Our size homeostasis experiments in the absence of Cdr2 also revealed additional layers of regulation: a secondary sizer control more closely based on volume and an adder- or timer-like control that operates in *cdr2*Δ cells born at a larger size. The secondary sizer mechanism could arise, for instance, from size scaling of Cdc25 expression [36], which is potentially volume based. Such multi-layer size control, also observed in [11], may provide a robust means of controlling cell size, able to withstand substantial perturbation.

This work highlights how cells can utilize different aspects of cell geometry in size regulation. Other related studies have shown how mammalian cells base their size control on surface area or volume [37] and how area to volume ratios can govern bacterial cell shape [38] and *Xenopus laevis* spindle size [39], and *E. coli* may control cell volume [40]. Evolution may have co-opted different geometric quantities for size regulation depending on the cell type. For instance, in plant cells, surface area may be more relevant because their interior is occupied by dynamic vacuoles. In contrast, the highly dynamic surface of migrating animal cells may make cytoplasmic volume a more reliable size indicator. Deciphering how molecules relay geometrical information will be key to understanding the mechanisms of size control.

## STAR★Methods

### Key Resources Table

**Table:** 

REAGENT or RESOURCE	SOURCE	IDENTIFIER
**Experimental Models: Organisms/Strains**		

*S. pombe* FC15: *h*^*-*^*WT (972)*	Lab collection	N/A
*S. pombe* FC2947: *h*^*-*^*rga2::ura4*^*+*^*ade6- leu1-32 ura4*^*-*^*D18*	Lab collection	N/A
*S. pombe* FC1901: *h*^*-*^*rga4::ura4*^*+*^*leu1-32 ura4*^*-*^*D18*	Lab collection	N/A
*S. pombe* FC3156: *h*^*+*^*cdr2-GFP:kanMX*	Lab collection	N/A
*S. pombe* FC3187: *h*^*+*^*rga2::ura4*^*+*^*cdr2-GFP:kanMX*	Lab collection	N/A
*S. pombe* FC3189: *h*^*-*^*rga4::ura4*^*+*^*cdr2-GFP:kanMX*	Lab collection	N/A
*S. pombe* FC3164: *h*^*-*^*mEGFP-cdr2-T166A ura4*^*+*^*D18*	This study	N/A
*S. pombe* FC3180: *h*^*-*^*rga2::ura4*^*+*^*mEGFP-cdr2-T166A*	This study	N/A
*S. pombe* FC3183: *h*^*-*^*rga4::ura4*^*+*^*mEGFP-cdr2-T166A*	This study	N/A
*S. pombe* FC3216: *h*^*-*^*cdr2-T166A*	Moseley Lab, JM2462	[4]
*S. pombe* FC3218: *h*^*-*^*cdr2-T166A rga2::ura4*^*+*^	This study	N/A
*S. pombe* FC3220: *h*^*-*^*cdr2-T166A rga4::ura4*^*+*^	This study	N/A
*S. pombe* FC3161: *h*^*+*^*cdr2::kanMX leu1-32*	This study	N/A
*S. pombe* FC3225: *h*^*-*^*cdr2:: kanMX rga2::ura4*^*+*^*leu1-32*	This study	N/A
*S. pombe* FC3227: *h*^*-*^*cdr2:: kanMX rga4::ura4*^*+*^*leu1-32*	This study	N/A
*S. pombe* FC2063: *h*^*-*^*pom1::natMX4 ade6- leu1-32 ura4-D18*	Lab collection	N/A
*S. pombe* FC3173: *h*^*-*^*ssp1-mEGFP::kanMX*	Moseley Lab, JM1260	[4]

**Software and Algorithms**		

FIJI ImageJ	NIH Image	[41]
Microbetracker	Jacobs-Wagner Lab	http://microbetracker.org/
DeepCell	Covert Lab	[30]
Morphometrics	Huang Lab	[29]
CellDataAnalysis.m	This study	Data S1

**Other**		

Cell Asic ONIX, 3.5 – 5.5 μm Y04C-02	EMD Millipore	N/A
Ti-Eclipse	Nikon Instruments	N/A
40X Ph2, 60X and 100X Ph3 Plan Apo objectives	Nikon Instruments	N/A
ILE; 561nm, 488nm, Borealis	Andor Technology	N/A
Zyla-4.2 sCMOS camera	Andor Technology	N/A
ImagEM EM-CCD camera (C9100-13)	Hamamatsu	N/A
CSU-10 spinning disk	Yokogawa	N/A
Dark panels environmental incubator	OkoLab	N/A

### Contact for Reagent and Resource Sharing

Request for resources and reagents should be directed to Lead Contact Martin Howard (Martin.Howard@jic.ac.uk).

### Experimental Model and Subject Details

Full genotypes of the strains used in this work are listed in the Key Resources Table. Standard methods for *S. pombe* growth and genetics were used [42]. Yeast cells were grown in YE5S rich medium with nutritional supplements at 175 mg/L. For solid media, 2% Difco Bacto agar was used. In general, strains were constructed using PCR-based homologous recombination methods for gene insertions in the yeast chromosome [43]. For genetic crosses, cells were mated and sporulated at 25°C on SPAS plates with supplements at 45 mg/L [44]. Spores were analyzed using tetrad dissection. Candidates were confirmed by PCR.

### Method Details

#### Imaging and image analysis

Yeast cells were generally grown at 25°C in rich YE5S media. Cultures were inoculated from single colonies into liquid YE5S media, grown overnight, diluted back and grown at least 6 hours to mid-exponential phase. For size homeostasis experiments, cells were introduced into microfluidic flow chambers (EMD Millipore, Cell Asic ONIX, 3.5 – 5.5 μm Y04C-02). Chambers were first primed for 15 min with pre-warmed media, after which cells were loaded at a 1:20 dilution. Fresh, warmed YE5S media was flowed into culture chambers at 5 psi at all time points. Cells were imaged in phase contrast in time-lapse every 10 min. Cell growth and division were analyzed after about 1 h introduction into the plate and followed for 2-3 generations. By analyzing each generation, we found that the cell size and division data were consistent for the duration of this imaging period. For fluorescence Cdr2 imaging, cells were grown to mid-exponential phase at 25°C at YE5S in a similar manner. Cells were concentrated in a mini-microfuge for 10 s and placed onto YE5S + 1% agarose pads, then sealed with valap. Multiple fields (up to 100 fields) were imaged within 30 min at 25°C. In order to measure the entire nodal signal for all the strains, we acquired z stacks of 19 slices with a spacing of 0.4 μm (z-resolution of the objective) (Figure S4E).

##### Microscopy

All imaging was performed on a dual spinning disk confocal and widefield microscope system consisting of a Ti-Eclipse (Nikon Instruments) stand with automated XYZ stage (ASI Instruments). Temperature was maintained by an environmental incubator (OkoLab), which was warmed for at least 1 h prior to imaging. Phase-contrast widefield imaging was performed with a 100X Ph3 Plan Apo objective (Nikon Instruments) and a Zyla-4.2 sCMOS camera (Andor Technology) with 2x2 binning (1x1 binning was used for higher pixel resolution imaging in Figure 4E, pixel size of 67 nm). For data in Figures 1E, 1F, S1E, S1F, S4A, and S4B, a 40X Ph2 Plan Apo objective (Nikon Instruments) with a 1.5x magnification tube lens was used. Fluorescence imaging was performed using a 60X Plan Apo objective (Nikon Instruments) with a solid-state laser source (Andor Technologies, ILE; 561nm, 488nm, Borealis), spinning disk confocal head (Yokogawa CSU-10) and EM-CCD camera (Hamamatsu).

##### Cell segmentation

For size homeostasis studies, phase contrast images were analyzed using a partially automated pipeline. First, images were pre-processed using FIJI (ImageJ) for data handling, where each cell was manually cropped at birth and division, as identified by initial cell division and presence of septa, respectively. Next, a deep neural network machine learning algorithm [30] was used to generate binary images for feature (outline/cytoplasm) identification. These contours were then used for traditional gradient segmentation in Morphometrics, a MATLAB-based software package that further implements routines for sub-pixel contour resolution [29]. Cells with an obvious incorrect segmentation were manually removed. High resolution image analysis in Figure 4E was aided by the large number (≈200) of radius measurements, allowing a highly precise estimate of each cell’s mean radius (see next section). Furthermore, the radius determination in each measurement was not limited by the close overlap between two closely positioned fluorescent peaks, meaning that the diffraction limit was not strongly constraining in this case. Manual segmentation was required for bright-field images taken with GFP acquisition. The tool Microbetracker was used to assist with this manual cell segmentation.

##### Cell geometry measurements

For a given cell segmentation, the cell symmetry axis was identified using principal component analysis of the cloud of points internal to the cell. Along this axis we measured the cell length *L*. The shortest distance from the border to the symmetry axis defined the profile *R*(*x*), 0 ≤ *x* ≤ *L*, of the cell radius. From *R*(*x*), and in order to confirm the robustness of our results, we calculated the surface area and volume of the cells of a given strain in three different ways: (1) by *rotation* of the *R*(*x*) function of each single cell around the symmetry axis (this approach avoids assuming a cylindrical shape of the cell), (2) by calculating the mean radius of each *single cell* and then employing the appropriate equations for surface area and volume of a cylinder with hemispherical ends; (3) by assuming that every cell of a given strain has the same cell radius (average over the *cell population*) and then using the same equations for area and volume of a cylinder with hemispherical ends (Table S1). Figures S1A, S1G, S2E, S2F, S3H, S3I, and S4C show that similar results were obtained with each method. Plots in the main text, except Figure 4E, report data obtained with methodology (3), as do all Supplemental Figures, except the bar charts in Figures S1A, S1G, S2E, S2F, S3H, S3I, and S4C. Figure 4E uses methodology (2). All calculations were performed in MATLAB (see Data S1).

##### Calculation of Cdr2 cytoplasmic concentration and Cdr2 nodal amount and density

For the cytoplasmic Cdr2 concentration, we used the mid-focal plane image and measured the averaged GFP fluorescence intensity in the cytoplasm (specifically excluding the nuclear region). In the calculation of the Cdr2 nodal amount from its GFP intensity, we used the following methodology. We used a sum projection (over the 19 slices of the z stack, Figure S4E). From this sum projection, we first identified and measured the nodal area as follows. Fluorescence intensity was summed and projected onto the cell symmetry axis. This procedure gave the profile of the Cdr2 intensity along the cell length (Figures S4E and S4F). The nodal peak was then fitted with a Gaussian profile (with mean *m* and variance σ^2^) that emerges from the “background” intensity from the rest of the membrane and cytoplasm. The width of the nodal area was then set equal to *W* = 4σ. The summation of the intensity in the range *m* ± 2σ gave the “Cdr2 nodal intensity.” The ratio between this nodal intensity and the area of the nodal region $\left(A_{nodal}=2\pi RW\right)$ gave the “Cdr2 nodal density.” This procedure was automated by implementing custom MATLAB code (see Data S1).

#### Mathematical model

We describe here the model we use to predict length scaling of the nodal Cdr2 density in *cdr2-T166A* mutant. In the following, [Ssp1] and [Cdr2_u_] denote the cytoplasmic concentrations of Ssp1 and unphosphorylated Cdr2, respectively, $k_{p}$ the kinetic constant for Cdr2 phosphorylation by Ssp1, $k_{b}$ the membrane-binding constant of unphosphorylated Cdr2, $N_{nodal}$ the total amount of Cdr2 in the nodal region, λ the Cdr2 nodal dissociation parameter and $N_{u}$ the number of copies of unphosphorylated Cdr2 in the cytoplasm.

##### Scaling of nodal Cdr2 density in wild-type cells

We first write an equation for the dynamics of the cytoplasmic population of unphosphorylated Cdr2, with protein copy number $N_{u}$. Unphosphorylated cytoplasmic Cdr2 can follow two pathways: either be phosphorylated in the cytoplasm by Ssp1 before membrane-binding (with overall rate $-\;k_{p}\left[Ssp1\right]\left[Cdr2_{u}\right]V_{cell}$), or alternatively undergo spontaneous membrane-binding (with overall rate $-k_{b}\left[Cdr2_{u}\right]A_{memb}$). Consistent with turnover of Cdr2 molecules within nodes [3, 17], Cdr2 can dissociate from nodes and return to the cytoplasm (with overall rate $+\lambda N_{nodal}$). The equation for $N_{u}$ is then:

$$\frac{dN_{u}}{dt}=\;-\;k_{p}\left[Ssp1\right]\left[Cdr2_{u}\right]V_{cell}-k_{b}\left[Cdr2_{u}\right]A_{memb}+\lambda N_{nodal}.$$

Following these processes, a non-nodal population of membrane Cdr2 exists which can subsequently relocate to the nodes, in an incompletely understood process. Nevertheless, since this latter process involves the membrane population of Cdr2, and does not directly affect cytoplasmic levels of Cdr2, it does not appear in this equation. For the same reason, we do not consider in detail the dynamics of node formation. Of course, this process may be important for downstream signaling or other purposes, but in principle is not itself required for size scaling. Cdr2 dynamics are in an approximate steady-state because of rapid Cdr2 nodal turnover ($t_{1/2}=3$ min, much shorter than the cell cycle period [3, 17]), and because nodal Cdr2 levels are unchanged with time in non-growing cells [3]. By setting $dN_{u}/dt=0$ (steady-state condition), we have:

(1) $$k_{p}\left[Ssp1\right]\left[Cdr2_{u}\right]V_{cell}+k_{b}\left[Cdr2_{u}\right]A_{memb}=\lambda N_{nodal}.$$

We then make the following two assumptions: (i) Ssp1-mediated phosphorylation occurs much faster than spontaneous membrane binding of unphosphorylated Cdr2; (ii) membrane binding of phosphorylated Cdr2 is also sufficiently rapid. Because of assumption (i), the term $k_{b}\left[Cdr2_{\text{u}}\right]A_{memb}$ can be ignored, and because of assumption (ii), the unphosphorylated cytoplasmic Cdr2 concentration can be approximated by the total cytoplasmic Cdr2 concentration [Cdr2]. To examine whether these assumptions are reasonable, we measured the Cdr2 membrane affinity, comparing the membrane (non-nodal) to cytoplasmic ratio of Cdr2 fluorescence density. This ratio was higher in the wild-type compared to the case of non-phosphorylatable Cdr2-T166A (see Figure S2K) consistent with these assumptions. Furthermore, we found experimentally that both Cdr2 and Ssp1 cytoplasmic concentrations are constant (Figures S2B, S2I, and S2J, respectively), so that $k_{p}\left[Ssp1\right]\left[Cdr2\right]$can be replaced by a simple factor K. Incorporating this finding leads to the following simple equation:

(2) $$KV_{cell}=\lambda N_{nodal},$$

which indicates that the Cdr2 nodal amount scales with volume, as observed experimentally. To deduce the Cdr2 nodal density (number of Cdr2 proteins per unit area of the nodal region), we divide $N_{nodal}$ by the area occupied by the nodes, $A_{nodal}=2\pi RW$, where W is the nodal region width. We found experimentally that W is approximately constant with respect to varying cell lengths and radii (Figure 2D). Since $V_{cell}\approx \pi R^{2}L,$ we find that the Cdr2 nodal density $\rho_{nodal}$ scales with area2πRL (Figure 2C equations), again as observed experimentally.

##### Prediction about a non-phosphorylatable Cdr2 mutant

This model provides a striking prediction that it may be possible to alter the scaling of the nodal density $\rho_{nodal}$ from area to length. Equation 1 has two terms: the first proportional to volume (Cdr2 phosphorylation by cytoplasmic Ssp1) and the second proportional to area (direct Cdr2 membrane binding). We previously ignored Cdr2 membrane binding by assuming fast Ssp1 phosphorylation. However, if we remove the cytoplasmic phosphorylation reaction ($k_{p}=0$ in Equation 1), we retain the surface area term:

(3) $$k_{b}\left[Cdr2\right]A_{memb}=\lambda N_{nodal}.$$

Therefore, in a mutant where Cdr2 is non-phosphorylatable by Ssp1 and has a constant cytoplasmic concentration, this equation predicts surface area scaling of total nodal Cdr2, and hence the nodal Cdr2 density should scale with length (Figure 3A equations).

##### The key step for the size scaling

The key to manipulating the geometrical size sensing of Cdr2 in this model lies in identifying where, after nodal unbinding, the protein next interacts: if this occurs in the cytoplasm, area sensing results, if this occurs on the membrane, length sensing results. More detailed Cdr2 models incorporating many of these additional processes were analyzed in depth providing a more detailed description of the Cdr2 nodal accumulation (e.g., the increase of Cdr2-pT166 levels while the cell elongates). Nevertheless, as expected, our fundamental size scaling results were unaffected, and we therefore omit these detailed analyses. Previous models of Cdr2 dynamics [3], have discussed size scaling dynamics in terms of “*antenna models*,” similar to those used in models of microtubule size scaling dynamics [45]. The antenna is the region over which size information is acquired by a molecule, before the molecule is itself concentrated into a spatially limited region for size readout. The models used here can also be cast in this form: for the wild-type (*cdr2-T166A*), the cytoplasm (membrane) is the “antenna” giving total Cdr2 intensity scaling with cell volume (surface area). When generating the local Cdr2 density, these quantities are divided by the nodal area, leading to cell surface area (length) size scaling.

##### Additional considerations

As mentioned above, the relatively rapid Cdr2 turnover within nodes (with a $t_{1/2}=3$ min), much faster than the cell cycle timescale, ensures that the Cdr2 dynamics come into steady-state. This timescale is consistent with previous FRAP experiments [3, 17], though these experiments did also reveal an immobile fraction, which is likely related to the internal part of each node. Nevertheless, since the size distribution of the nodes (as given by fluorescence intensity of individual nodes using mEGFP-labeled Cdr2) does not change with cell length [3], the immobile part is always a constant fraction f of the total Cdr2 nodal amount. Therefore, replacing $N_{nodal}$ with the mobile fraction $N_{mobile}=N_{nodal}-N_{immobile}=\left(1-f\right)\cdot N_{nodal}$ within our model again made no difference to the size scaling dynamics. Equations 1 and 2 in the main text also incorporate the entire interior of the cell as locations where Cdr2 can be phosphorylated by Ssp1. However, it is worth noticing that only a fraction of the cytoplasmic volume is accessible to Cdr2, i.e., the total volume reduced by the nucleus and the volume of cytoplasmic vacuoles. Clearly, the two regions are not equivalent. Nevertheless, it has been shown previously that there exists a constant ratio between the nuclear volume and cell volume [3, 46]. Moreover, we have verified that a similar result applies to the vacuoles (Figures S2I and S2L). Consequently, the volume of the accessible cytoplasm is a constant fraction of the entire cell volume (i.e., $V_{accessible}=c\cdot V_{cell}$) and therefore Equations 1 and 2 are still valid. A similar result has been obtained in budding yeast [47]. The vacuole versus cell volume ratio was calculated based on the cross-sectional areas found at the mid focal plane (Figure S2I). We assumed here that the total vacuole cross-sectional area fraction in other focal planes is the same as at the mid focal plane.

### Quantification and Statistical Analysis

#### Normalized RMSD calculations and p values

To calculate the normalized RMSD (Root Mean Square Deviation) for a set of three strains, we used the following methodology. The calculation was performed on the regression lines of the binned data. First, we identified a range on the x axis that was approximately in common between the three strains. For each pair of strains, we calculated the RMSD over this interval (discretized with a set of equally spaced points$,\;{\left\{x_{i}\right\}}_{i=1,..,N},\;N=20$), i.e., $RMSD\left(y,y^{'}\right)=1/N\sqrt{\sum_{i}{\left[y\left(x_{i}\right)-y^{'}\left(x_{i}\right)\right]}^{2}}$. The sum of all these RMSDs between all three pairs of strains was then divided by the mean of all the y values.

Since the calculation is performed on the common x axis range, the RMSD only quantifies the overlap in the y direction, as required. However, when the three strains (thin, wild-type and fat) share only a narrower overlap range along the x axis, a visual impression of a weaker overlap may appear. An example is the volume scaling of the total Cdr2 nodal fluorescence in Figure 2A: the fact that cells divide at a constant area reduces the overlap in the x direction in the rightmost plot which reports volume. A similar effect occurs in Figure 3C in the case of Cdr2-T166A nodal density versus length. The RMSD value overcomes this problem and provides an appropriate quantification of the overlap. We also tested for the significance of the difference between the RMSDs for two geometrical quantities. Linear regression provided the slope and intercept with the standard deviation. From this statistical information, we numerically derived the distribution of the RMSD for each geometrical quantity. We then used t tests to compute p values for the RMSDs to be different.

#### Generalized size measure analysis

In addition to our analysis to distinguish between the three standard geometrical quantities (length, area and volume), we also used the data to analyze more general and unbiased measures of cell size, as we describe below. In Figure S1B (size homeostasis in *rga2*Δ, wild-type and *rga4*Δ), we investigate the generalized size measure $R^{\gamma }L$, asking what value of γ would give the smallest normalized RMSD with our experimental data. This procedure allows us to compare measures of size different from the standard length, area and volume without any bias. We find that an optimum is achieved for γ≈1, i.e., for surface area sensing, $A_{cell}\propto RL$. In Figures S1H and S4D, we repeat this analysis for size homeostasis in *cdr2*Δ *rga2*Δ, *cdr2*Δ, *cdr2*Δ *rga4*Δ, and in *cdr2-T166A rga2*Δ, *cdr2-T166A*, *cdr2-T166A rga4*Δ, respectively. We now find that the smallest RMSDs are achieved for γ≈1.62 and γ≈1.66, respectively. These results are close to cell volume sensing, since $V=\pi R^{2}\left(L-2R/3\right)$, which can be approximated as$\;V\approx \pi R^{2}L^{\theta }$, with θ>1, or $V\approx \pi {\left(R^{\gamma }L\right)}^{2/\gamma }$, with an effective exponent of γ≈1.75. Here and in all figures referred to in this section we use the cell population average methodology (Table S1) for segmentation. In Figures S2C and S2D, we fit the experimental total nodal mEGFP-Cdr2 intensity and nodal mEGFP-Cdr2 density from pooled *rga2*Δ, wild-type and *rga4*Δ data against the general size measure $R^{\alpha }L^{\beta }$. We again search for optimal respective values of α/β with minimal RMSD as compared to our experimental data, allowing us to probe how the Cdr2 levels scale with generalized measures of cell size other than length, area and volume. We find optimal values of around α/β≈2 for the total nodal Cdr2, consistent with volume scaling, while for the nodal Cdr2 density α/β≈1 was optimal, consistent with area scaling. In Figures S3D and S3E, we repeat this analysis for size homeostasis in *cdr2-T166A rga2*Δ, *cdr2-T166A*, *cdr2-T166A rga4*Δ. We now find optimal values of around α/β≈1 for the total nodal Cdr2, consistent with area scaling, while for the nodal Cdr2 density α/β≈0 was optimal, consistent with length scaling.

### Data and Software Availability

MATLAB code CellDataAnalysis.m (see Data S1) reads the segmentation output from Microbetracker/Morphometrics and calculates the cell geometry features (radius, length, surface area and volume according to expressions in Table S1). By using this segmentation data and the fluorescence images (mEGFP-Cdr2 signal), the code also calculates the nodal intensity, nodal density and cytoplasmic level of Cdr2 (as described in the paragraph “Calculation of Cdr2 cytoplasmic concentration and Cdr2 nodal amount and density” above).

## Supplemental Information

Document S1. Figures S1–S4 and Table S1

Data S1. MATLAB Code for the Calculation of Cell Geometry Features and Analysis of Fluorescence Data, Related to STAR Methods

Document S2. Article plus Supplemental Information
