# Supplementary material for: Reprogramming Cdr2-Dependent Geometry-Based Cell Size Control in Fission Yeast
Source: Curr Biol. 2019 Jan 21;29(2):350–358.e4. doi: 10.1016/j.cub.2018.12.017 (PMC6345630; doi:10.1016/j.cub.2018.12.017)
Supplement: Document S1. Figures S1–S4 and Table S1 [file mmc1.pdf]

Current Biology, Volume *29*

Supplemental Information

Reprogramming Cdr2-Dependent Geometry-Based Cell Size Control in Fission Yeast

Giuseppe Facchetti, Benjamin Knapp, Ignacio Flor-Parra, Fred Chang, and Martin Howard

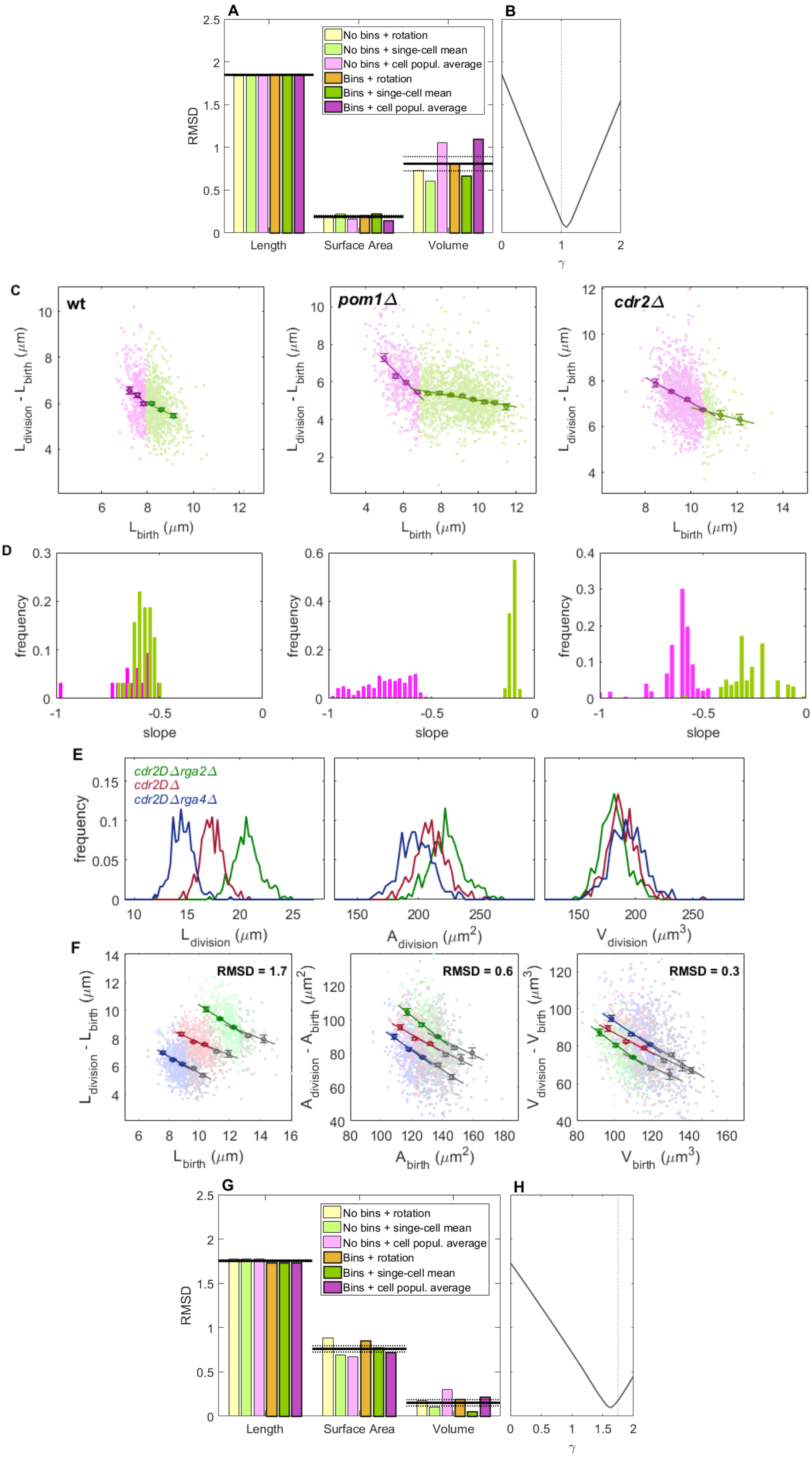
A
B
C
D
E
F
G
H
No bins + rotation
No bins + singe-cell mean
No bins + cell popul. average
Bins + rotation
Bins + singe-cell mean
Bins + cell popul. average
RMSD
Length
Surface Area
Volume
wt
pom1Δ
cdr2Δ
frequency
slope
cdr2DΔrga2Δ
cdr2DΔ
cdr2DΔrga4Δ
RMSD = 1.7
RMSD = 0.6
RMSD = 0.3

Figure S1. Size homeostasis in the wild-type and *cdr2*Δ mutant. Related to Figure 1.

(**A**) Robustness to changes in the methodology for calculating the area or volume: we calculated the surface area and volume of the cells of a given strain in three different ways: (1) by *rotation* of the *R*(*x*) function (the shortest distance from the cell border to the symmetry x-axis) of each single cell around the symmetry axis (this approach avoids assuming a cylindrical shape of the cell); (2) by calculating the mean radius of each *single cell* and then employing the appropriate equations for surface area and volume of a cylinder with hemispherical ends; (3) by assuming that every cell of a given strain has the same cell radius (average over the *cell population*) and then using the same equations for area and volume of a cylinder with hemispherical ends (see Table S1). Bars report Normalized Root Mean Square Deviation (RMSD, divided by mean value) from equivalents of plots in Figure 1D for cell length, area and volume, but where cell geometry is calculated with each of the above three possible methods, with and without bin analysis: smallest RMSD always obtained for surface area. Black lines: average over the 6 methodologies with dotted lines indicating ± standard error.

(**B**) Analysis for generalised size measure $R^{\gamma}L$: smallest normalized RMSD (calculated as in Figure 1D) is achieved for $\gamma \approx 1$, i.e. for surface area sensing, $A_{\text{cell}} \propto RL$.

(**C**) Because of their longer division size, *cdr2*Δ mutants might show two regimes in a size homeostasis plot: a steeper part (sizer-like) for shorter sizes at birth (lower than a given threshold, magenta colour), and a flatter part for longer sizes at birth (higher than the threshold, green colour). Example of absence of two regimes in wild-type cells (left panel, FC15, n=1061, data from Figure 1D). Example of two regimes in the well-known case of *pom1*Δ mutant (middle panel, FC2063, n=1802). Two regimes in *cdr2*Δ mutant (right panel, FC3161, n=1277, data from Figure 1F). Binned data (with mean ± standard error) and associated regression lines shown. The threshold value between the two regimes was fixed at 60% of the average division length.

(**D**) In order to identify the two regimes more precisely, we scanned different positions of the threshold and calculated the slope of the two subsets of cells. We found that the largest difference between the two slopes is where the threshold is at about 60% of the division size. We report here the histogram analysis on the two slopes when this border is scanned over a range from about 50% to 70%. The wild-type and *pom1*Δ mutant are used as negative and positive references, respectively. Left panel: histogram distribution of the slopes for wild-type. Middle panel: histogram distribution of the slopes for *pom1*Δ. Right panel: histogram distribution of the slopes for *cdr2*Δ. Slope values around -0.6 (close to those found for the wild-type) for the steeper part in *cdr2*Δ suggest that a sizer mechanism is still operating.

(**E**) Repeated experiment of *cdr2*Δ size sensing. Distribution of cell length, surface area and volume at division for *cdr2*Δ *rga2*Δ, *cdr2*Δ and *cdr2*Δ *rga4*Δ, as in Figure 1E. Colour legend: *cdr2*Δ *rga2*Δ (FC3225, green, n=932), *cdr2*Δ (F3161, red, n=919) and *cdr2*Δ *rga4*Δ (FC3227, blue, n=1034).

(**F**) Size homeostasis plots from repeated experiment for *cdr2*Δ *rga2*Δ, *cdr2*Δ and *cdr2*Δ *rga4*Δ using cell length, surface area or volume as size measure, as in Figure 1F. Slopes for shorter sizes at birth only (less than 60% of the average division size, i.e. for the sizer regime, coloured lines; see panel C) are -0.7 (*cdr2*Δ *rga2*Δ), -0.6 (*cdr2*Δ) and -0.6 (*cdr2*Δ *rga4*Δ). Slopes of the regime for longer sizes at birth (grey lines) are -0.5, -0.2 and -0.6, respectively. Binned data (with mean ± standard error) and associated regression lines shown. Normalized Root Mean Square Deviation (RMSD, divided by mean value) also stated. The t-test on the RMSD of volume vs RMSD of area shows a *p*-value lower than 10^{-20}.

(**G**) Bars report normalized RMSD from equivalents of plots in Figure 1F, but where cell geometry is calculated with each of the three possible methods from Table S1, with and without bin analysis. Smallest RMSD always obtained for volume. Black lines: average over the 6 methodologies, with dotted lines indicating ± standard error.

(**H**) Analysis of data from Figure 1F for generalised size definition, $R^{\gamma}L$: smallest RMSD (calculated as in Figure 1F) is achieved for $\gamma \approx 1.62$. This result is close to cell volume sensing (since $V = \pi R^2(L - 2R/3)$, the theoretical value is $\gamma \approx 1.75$, dotted line; see STAR Methods). Numerical analysis showed that this shift toward volume sensing is not due to the increase in the division length (data not shown).

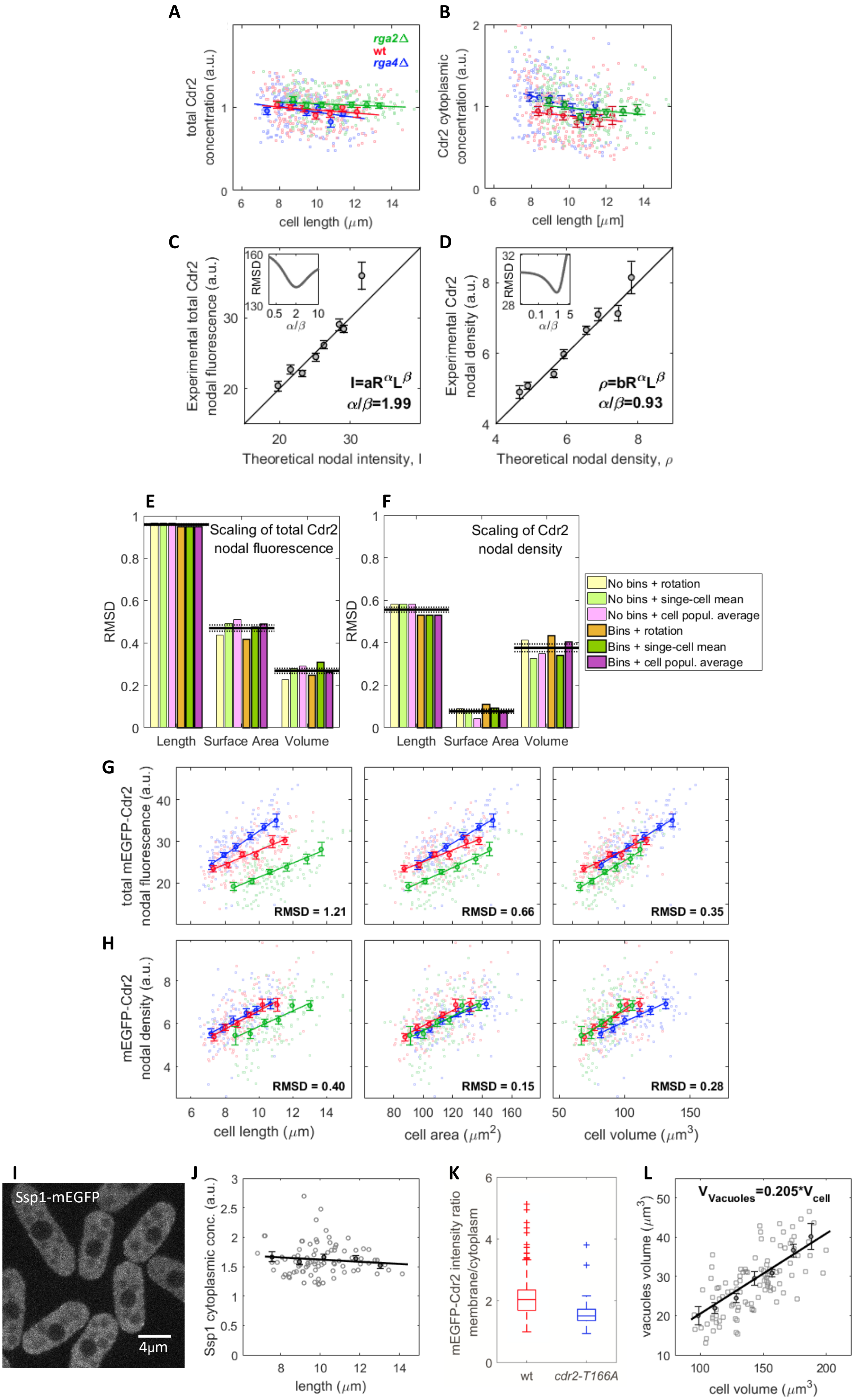

A
total Cdr2 concentration (a.u.)
rga2Δ
wt
rga4Δ
cell length (μm)
B
Cdr2 cytoplasmic concentration (a.u.)
cell length [μm]
C
Experimental total Cdr2 nodal fluorescence (a.u.)
RMSD
α/β
I=aR^αL^β
α/β=1.99
Theoretical nodal intensity, I
D
Experimental Cdr2 nodal density (a.u.)
ρ=bR^αL^β
α/β=0.93
Theoretical nodal density, ρ
E
Scaling of total Cdr2 nodal fluorescence
RMSD
Length
Surface Area
Volume
F
Scaling of Cdr2 nodal density
No bins + rotation
No bins + singe-cell mean
No bins + cell popul. average
Bins + rotation
Bins + singe-cell mean
Bins + cell popul. average
G
total mEGFP-Cdr2 nodal fluorescence (a.u.)
RMSD = 1.21
RMSD = 0.66
RMSD = 0.35
H
mEGFP-Cdr2 nodal density (a.u.)
RMSD = 0.40
RMSD = 0.15
RMSD = 0.28
cell length (μm)
cell area (μm²)
cell volume (μm³)
I
Ssp1-mEGFP
4μm
J
Ssp1 cytoplasmic conc. (a.u.)
length (μm)
K
mEGFP-Cdr2 intensity ratio membrane/cytoplasm
wt
cdr2-T166A
L
V_Vacuoles=0.205*V_cell
vacuoles volume (μm³)
cell volume (μm³)

Figure S2. Size scaling behaviour of Cdr2. Related to Figure 2.
(**A-B**) Total EGFP-Cdr2 fluorescence divided by cell volume as function of cell length for *rga2*Δ, wild-type and *rga4*Δ (panel A). Plot of mEGFP-Cdr2 cytoplasmic concentration measured as mean cytoplasmic fluorescence intensity (excluding nuclear region) as function of cell length for *rga2*Δ, wild-type and *rga4*Δ (panel B).
(**C-D**) Fit of experimental total nodal mEGFP-Cdr2 intensity (from Figure 2A) and nodal mEGFP-Cdr2 density (from Figure 2B) from pooled *rga2*Δ, wild-type and *rga4*Δ data against general size measure $R^{\alpha}L^{\beta}$, searching for optimal respective values of α/β with minimal RMSD (insets). Optimal values stated in panels: for total intensity, $\alpha/\beta \approx 2$ was optimal, consistent with volume scaling (panel C), for density, $\alpha/\beta \approx 1$ was optimal, consistent with area scaling (panel D).
(**E-F**) Bars report results using each of the methods from Table S1, with and without bin analysis. (panel E) Normalized RMSD of mEGFP-Cdr2 nodal intensity plotted against cell length, surface area and cell volume, from equivalent plots as in Figure 2A. Cell volume always shows the smallest RMSD. (panel F) Normalized RMSD of Cdr2 nodal density plotted against cell length, surface area or volume, from equivalent plots as in Figure 2B. Surface area always shows the smallest RMSD. Black lines: average over the 6 methodologies with dotted lines indicating ± standard error.
(**G-H**) Repeated experiment as in Figure 2A-B. Plots of total nodal intensity of mEGFP-Cdr2 (top row) and of nodal density of mEGFP-Cdr2 (bottom row) for *rga2*Δ, wild-type and *rga4*Δ as function of length, surface area and volume. Colour legend: *rga2*Δ (FC3187, green, n=150), wild-type (FC3156, red, n=151) and *rga4*Δ (FC3189, blue, n=140). Normalised Root Mean Square Deviation (RMSD between binned data) also stated; t-tests on the differences between the RMSDs give *p*-values < 10^{-8}.
(**I**) Mid-focal plane confocal image on agar of cells expressing Ssp1-mEGFP (FC3173).
(**J**) Cytoplasmic concentration of Ssp1 measured as mean cytoplasmic fluorescence intensity (excluding nuclear region). Strain FC3173, n=87.
(**K**) Membrane affinity measured as ratio of the mEGFP-Cdr2 mean intensity between membrane (non-nodal) and cytoplasm (from middle plane section image). For each box, the central mark indicates the median; the bottom and top edges of the box indicate the 25th and 75th percentiles, respectively; the whiskers extend to the most extreme data points not considered outliers; outliers are reported individually by the '+' symbol. Stains: wild-type (FC3156, n=224), *cdr2-T166A* (FC3164, n=257).
(**L**) Proportionality between total vacuole volume and cell volume. We assumed that the round dark objects in the images represent the nucleus (large medial structure) and vacuoles (smaller structures). From a single mid-focal plane image, we estimated total vacuole volume and cell volume (see STAR Methods for details). Fitted line has the reported equation. These data suggest that total vacuole volume scales proportionally with cell volume. Strain FC3173, n=103.
Binned data (with mean ± standard error) and associated regression line also shown in all panels (except E, F, I, K).

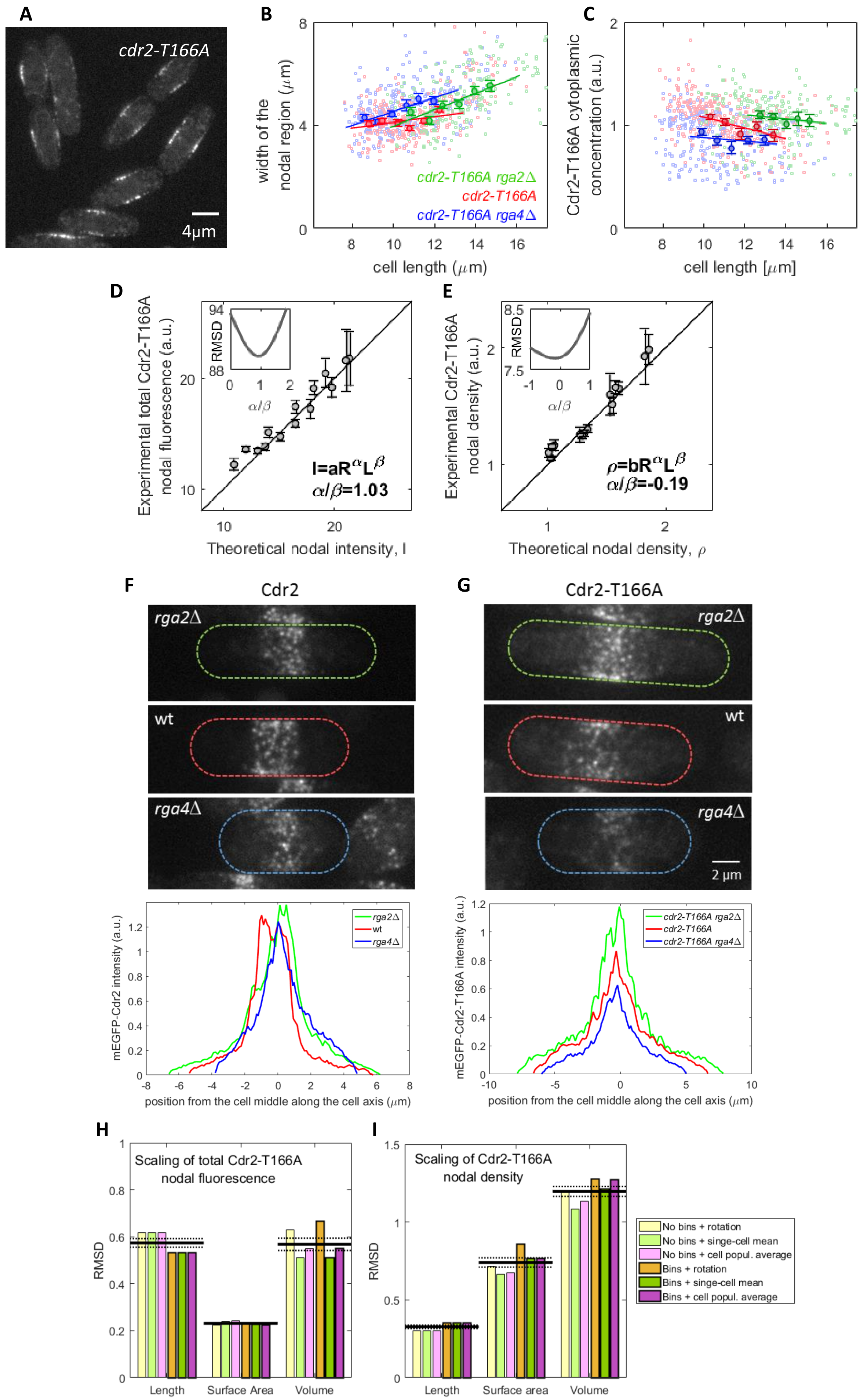

A
cdr2-T166A
4μm
B
width of the nodal region (μm)
cell length (μm)
cdr2-T166A rga2Δ
cdr2-T166A
cdr2-T166A rga4Δ
C
Cdr2-T166A cytoplasmic concentration (a.u.)
cell length [μm]
D
Experimental total Cdr2-T166A nodal fluorescence (a.u.)
Theoretical nodal intensity, I
RMSD
α/β
I=aR^αL^β
α/β=1.03
E
Experimental Cdr2-T166A nodal density (a.u.)
Theoretical nodal density, ρ
ρ=bR^αL^β
α/β=-0.19
F
Cdr2
rga2Δ
wt
rga4Δ
G
Cdr2-T166A
2 μm
mEGFP-Cdr2 intensity (a.u.)
position from the cell middle along the cell axis (μm)
mEGFP-Cdr2-T166A intensity (a.u.)
cdr2-T166A rga2Δ
cdr2-T166A
cdr2-T166A rga4Δ
H
Scaling of total Cdr2-T166A nodal fluorescence
RMSD
Length
Surface Area
Volume
I
Scaling of Cdr2-T166A nodal density
Surface area
No bins + rotation
No bins + singe-cell mean
No bins + cell popul. average
Bins + rotation
Bins + singe-cell mean
Bins + cell popul. average

Figure S3. Size scaling behaviour of Cdr2-T166A. Related to Figure 3.
(**A**) Confocal image of Cdr2-T166A fluorescence at the middle plane.
(**B-C**) Width of the nodal area does not change with cell radius but varies weakly with cell length, i.e. $W \propto L^{\theta}, \theta \approx 0.4$ (panel B). Because of this, the Cdr2 nodal density scales with respect to cell length in a sublinear manner: $\rho_{\text{nodal}} \propto \frac{A_{memb}}{A_{nodal}} = \frac{2\pi RL}{2\pi RW} = \frac{2\pi RL}{2\pi RL^{\theta}} \approx L^{0.6}$. Nevertheless, these equations show that the independence of ρ_{nodal} from cell radius is still preserved. Hence, we expect ρ_{nodal} to scale much more closely with cell length than with either surface area or volume, as found in Figure 3. Cytoplasmic concentration of Cdr2-T166A measured as mean cytoplasmic fluorescence intensity (excluding nuclear region) does not significantly change with cell size (panel C). Colour legend: *cdr2-T166A rga2*Δ (FC3180, green, n=265), *cdr2-T166A* (FC3164, red, n=257) and *cdr2-T166A rga4*Δ (FC3183, blue, n=206). Measurements on same cells as used for Figure 3B,C. Binned data (with mean ± standard error) and associated regression lines also shown in (B,C).
(**D-E**) Fit of experimental total nodal mEGFP-Cdr2-T166A intensity (panel D) and nodal mEGFP-Cdr2-T166A density (panel E) from pooled *cdr2-T166A rga2*Δ, *cdr2-T166A* and *cdr2-T166A rga4*Δ data against general size measure $R^{\alpha}L^{\beta}$, searching for optimal respective values of α/β with minimal RMSD (insets). Optimal values stated in panels: for total intensity, $\alpha/\beta \approx 1$ was optimal, consistent with area scaling (Figure 3B), for density, $\alpha/\beta \approx 0$ was optimal, consistent with length scaling (Figure 3C).
(**F-G**) Image (sum projection) and fluorescence intensity profile along cell axis (same procedure as in Figure S4E) of three representative cells expressing wild-type mEGFP-Cdr2 (unmodified scaling, panel F) and of three representative cells expressing mEGFP-Cdr2-T166A (altered scaling, panel G). In each case, the three cells shown have approximately the same volume. Note that while mEGFP-Cdr2 intensity is similar in the three cells, mEGFP-Cdr2-T166A intensity is higher in thinner cells than fatter cells. These results are consistent with total Cdr2 nodal intensity scaling with volume in the wild-type, but with surface area in *cdr2-T166A* mutants, as predicted by our model.
(**H-I**) Scaling of mEGFP-Cdr2 total intensity and density in c*dr2-T166A rga2*Δ, *cdr2-T166A* and *cdr2-T166A rga4*Δ are not affected by data analysis methodology. Bars report results using each of the methods from Table S1, with and without bin analysis. Panel H: normalized RMSD of mEGFP-Cdr2-T166A total nodal intensity plotted against cell length, surface area and cell volume, from equivalent plots as in Figure 3B. Surface area always shows the smallest RMSD. Panel I: normalized RMSD of mEGFP-Cdr2-T166A nodal density plotted against cell length, surface area and cell volume, from equivalent plots as in Figure 3C. Cell length always shows the smallest RMSD. Black lines: average over the 6 methodologies with dotted lines indicating ± standard error.

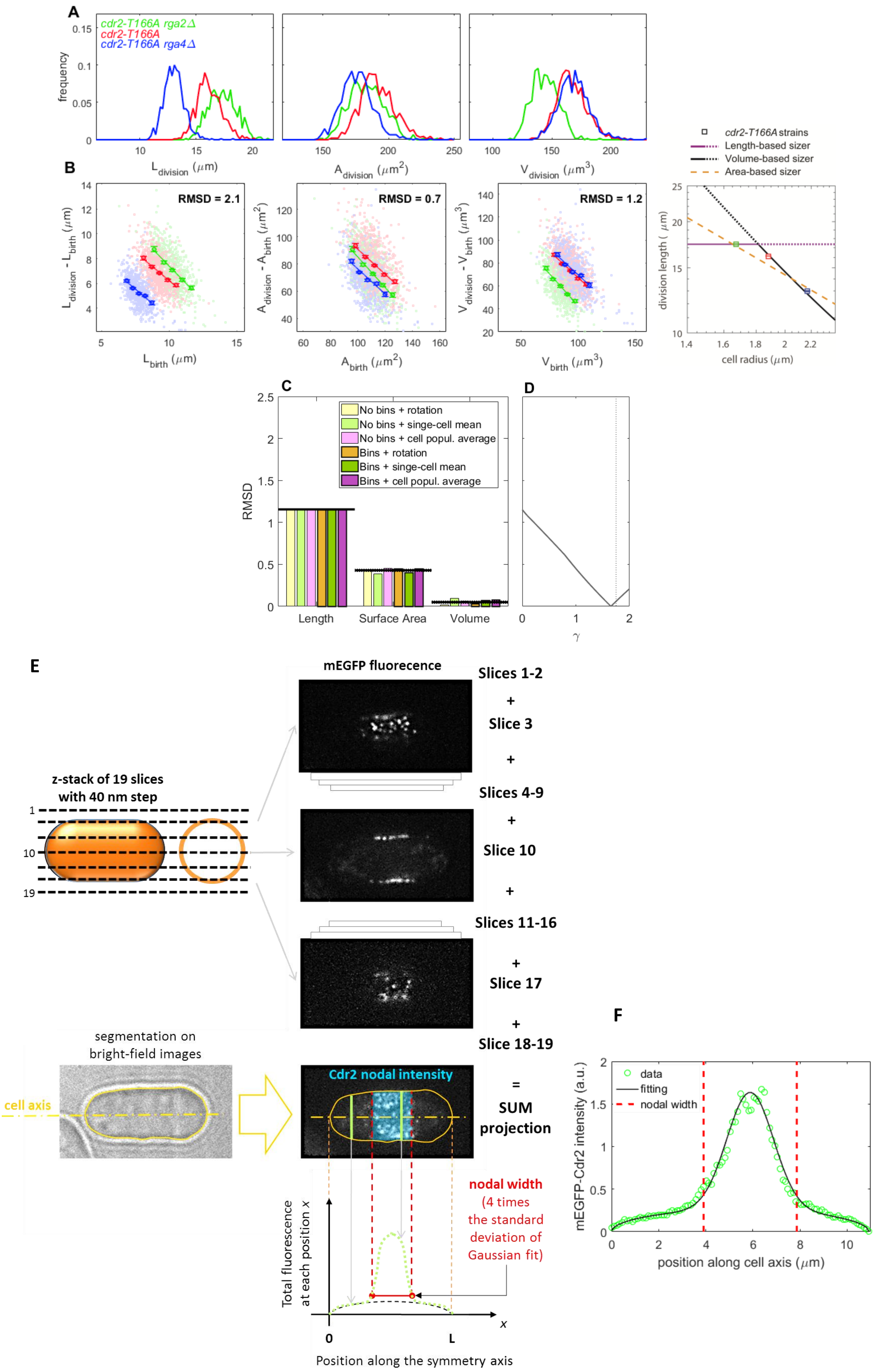

A
cdr2-T166A rga2Δ
cdr2-T166A
cdr2-T166A rga4Δ
frequency
L division (μm)
A division (μm2)
V division (μm3)
B
RMSD = 2.1
RMSD = 0.7
RMSD = 1.2
L division - L birth (μm)
A division - A birth (μm2)
V division - V birth (μm3)
L birth (μm)
A birth (μm2)
V birth (μm3)
cdr2-T166A strains
Length-based sizer
Volume-based sizer
Area-based sizer
division length (μm)
cell radius (μm)
C
No bins + rotation
No bins + singe-cell mean
No bins + cell popul. average
Bins + rotation
Bins + singe-cell mean
Bins + cell popul. average
RMSD
Length
Surface Area
Volume
D
γ
E
mEGFP fluorecence
Slices 1-2
+
Slice 3
+
Slices 4-9
+
Slice 10
+
Slices 11-16
+
Slice 17
+
Slice 18-19
=
SUM projection
z-stack of 19 slices
with 40 nm step
1
10
19
segmentation on
bright-field images
cell axis
Cdr2 nodal intensity
nodal width
(4 times
the standard
deviation of
Gaussian fit)
Total fluorescence
at each position x
0
L
x
Position along the symmetry axis
F
data
fitting
nodal width
mEGFP-Cdr2 intensity (a.u.)
position along cell axis (μm)

Figure S4. Size homeostasis in *cdr2-T166A* and image analysis methodology. Related to Figure 4 and STAR Methods.
(**A-B**) Repeated experiment for *cdr2-T166A*. Top panels: distribution of cell length, surface area and volume at division; Bottom panels: Size homeostasis plots using cell length, surface area or volume as size measure, for *cdr2-T166A rga2*Δ, *cdr2-T166A* and *cdr2-T166A rga4*Δ. Slopes are –1.0, -0.9 and -0.8, respectively. Colour legend *cdr2-T166A rga2*Δ (FC3218, green, n=596), *cdr2-T166A* (FC3216, red, n=902) and *cdr2-T166A rga4*Δ (FC3220, blue, n=750). Binned data (with mean ± standard error) and associated regression line shown. Normalized Root Mean Square Deviation (RMSD, divided by mean value) also stated. Last plot of panel B shows the same analysis as Figure 4D but for this repeated experiment. Data consistent with thinner *cdr2-T166A rga2*Δ cells dividing according to length, while wider *cdr2-T166A* and *cdr2-T166A rga*4Δ cells divide more closely specified by volume.
(**C**) Result for size homeostasis for *cdr2-T166A* and *cdr2-T166A rga4*Δ being more closely specified by volume (data from Figure 4A-B) is not affected by data analysis methodology. Bars report normalized RMSD from equivalents of plots such as those in Figure 4B, but where cell geometry is calculated with each of the three possible methods from Table S1, with and without bin analysis: smallest RMSD always obtained for volume. Black lines: average over the 6 methodologies with dotted lines indicating ± standard error.
(**D**) Analysis of *cdr2-T166A* and *cdr2-T166A rga4*Δ data from Figure 4B for generalised size definition, $R^{\gamma}L$: smallest RMSD (calculated as in Figure 1F) is achieved for $\gamma \approx 1.66$. This result is close to cell volume sensing (since $V = \pi R^2(L - 2R/3)$, the theoretical value is $\gamma \approx 1.75$, dotted line; see STAR Methods and Figure S1H).
(**E**) mEGFP-Cdr2 intensity profile (green dotted line) is a second sum projection along the cell axis. The profile is fitted by a Gaussian distribution above a background level (ellipse equation, dashed black line). Mean value and standard deviation of the Gaussian fit define the nodal region position and width (red segment), respectively. mEGFP-Cdr2 total nodal intensity is calculated as the total fluorescence in this region (blue shaded area).
(**F**) Example of mEGFP-Cdr2 intensity profile and fitting.

Name	"rotation"	"single-cell mean"	"cell population average"
Description	*Rotation around the symmetry axis of each single cell*	*Using mean radius of each single cell*	*Using average over entire cell population of mean radius of each single cell, with each strain considered separately*
Cell radius	Function $R(x)$	$\bar{R} = \frac{1}{L-2\delta}\int_{\delta}^{L-\delta} R(x)dx$	$\langle\bar{R}\rangle = \frac{1}{N}\sum_{i=1}^{N}\bar{R}_i$
Cell length	L	L	L
Cell surface area	$2\pi\int_0^L R(x)\sqrt{1+R'(x)^2}dx$	$2\pi\bar{R}L$	$2\pi\langle\bar{R}\rangle L$
Cell volume	$\pi\int_0^L R^2(x)dx$	$\pi\bar{R}^2L - \frac{2}{3}\pi\bar{R}^3$	$\pi\langle\bar{R}\rangle^2L - \frac{2}{3}\pi\langle\bar{R}\rangle^3$

Table S1. Three methods for calculating geometrical quantities for cells of a given strain. Related to STAR Methods. To test the validity of methods for calculating geometric quantities from mid-focal plane phase images of rod-shaped *S. pombe* cells, we compared these three methods. Figures S1A, G, S2E-F, S3H-I and S4C show these three methods lead to the same conclusions. The main figures (except Figure 4E, for which it is necessary to use "single-cell mean") report on data that were obtained with methodology "cell population average". Note that δ is approximated as *R*(*L*/2) for "cell radius".
